# In vivo bactericidal effect of colistin–linezolid combination in a murine model of MDR and XDR *Acinetobacter baumannii* pneumonia

**DOI:** 10.1038/s41598-020-74503-0

**Published:** 2020-10-15

**Authors:** Xiao-Lin Ma, Yong-Zhong Guo, Yan-Min Wu, Wei-Tao Gong, Jie Sun, Zhen Huang

**Affiliations:** 1 Department of Neurology, XuZhou Central Hospital, The Xuzhou School of Clinical Medicine of Nanjing Medical University, XuZhou Clinical School of Xuzhou Medical University, Xuzhou, 221009 Jiangsu China; 2 Department of Respiratory and Critical Care Medicine, XuZhou Central Hospital, The Xuzhou School of Clinical Medicine of Nanjing Medical University, XuZhou Clinical School of Xuzhou Medical University, Xuzhou, 221009 Jiangsu China

**Keywords:** Infectious diseases, Antimicrobials, Bacteria, Clinical microbiology

## Abstract

Recently, paradoxical combinations of colistin with anti-Gram-positive bacterial agents were introduced as a treatment alternative for multidrug-resistant *Acinetobacter baumannii* (MDRAB) infection. We assessed the therapeutic efficacy of the colistin–linezolid combination regimen in vitro and in a murine model of *Acinetobacter baumannii* pneumonia. A multidrug-resistant clinical strain (MDRAB31) and an extensively drug-resistant clinical strain (XDRAB78) were used in this study. The survival rates of mice and bacterial counts in lung tissue were used to assess the effects of colistin–linezolid combination. The survival rates of colistin–linezolid combination groups significantly increased compared with colistin groups for MDRAB31 (72% versus 32%, *P* = 0.03) and for XDRAB78 (92% versus 68%, *P* = 0.031). The colistin–linezolid combination groups significantly reduced the bacterial counts in lung tissue compared with colistin groups for MDRAB31 and for XDRAB78 *(P* < 0.05). The colistin–linezolid combination had a bactericidal and synergistic effect compared with colistin alone in time-kill assay and in murine model of pneumonia. Our data demonstrated the synergistic effect of colistin–linezolid combination regimen as a treatment alternative for the severe pulmonary infection caused by MDRAB and XDRAB.

## Introduction

*Acinetobacter baumannii* (*A. baumannii*) has become an important cause of hospital-associated infections affecting critically ill patients all over the world over the last decades^1^, and is mainly responsible for hospital-associated and ventilator-associated pneumonia^2^. The number of drugs that retain activity against *A. baumannii* has dramatically reduced for the global widespread of multidrug-resistant *A. baumannii* (MDRAB) and extensively drug-resistant *A. baumannii* (XDRAB) in hospital environment^3^. Since it is difficult to develop new classes of antibiotics, colistin is considered the last alternative for the treatment of MDR strains, and has been used increasingly^4^. However, for the sake of its heteroresistance, lipopolysaccharide modification, low plasma concentrations and toxicity, colistin monotherapy should be avoided^5^. Consequently, colistin-based combination therapies of existing drugs, including paradoxical combinations of colistin with anti-Gram-positive bacterial agents, have been made exploration and research to combat MDR *A. baumannii* infection^6–11^.

Since linezolid natural resistance to Gram-negative bacteria is due to the inability of drug to achieve effective intracytoplasmic concentrations, colistin can be used to increase the accumulation of linezolid by disrupting the permeability barrier of outer membrane and by inhibiting bacterial efflux pumps activities^12^. In a recent study, positive results of in vitro colistin–linezolid combination have been demonstrated in *Pseudomonas aeruginosa*, *Escherichia coli* and *A. baumannii*^12–15^.

Concurrently, little in vivo data exists concerning the effectiveness of colistin with linezolid for the treatment of *A. baumannii* infection. Since linezolid has been approved mainly for the hospital-acquired pneumonia treatment, our objective was to investigate the efficacy of the combination of colistin with linezolid in a murine model of *A. baumannii* pneumonia.

## Results

### Susceptibility tests and checkerboard assays

The MICs results of the colistin, linezolid and colistin–linezolid combination in *A. baumannii* are shown in Table 1. Both strains of *A. baumannii* resulted susceptible to colistin and showed high values to linezolid. The colistin–linezolid combination resulted in synergy for MDRAB31, additivity/indifference for XDRAB78.

**Table 1:** The results of MICs and checkerboard assays for *A. baumannii* strains and control strain.

Strain	MIC (mg/ml)			FICI
	COL	LNZ	COL/LNZ	LNZ + COL
MDRAB 31	0.5	> 256	0.125/8	0.281
XDRAB 78	0.5	> 256	0.25/8	0.531
PA ATCC 27853	0.5	> 256	0.25/64	0.75

*COL* colistin, *LNZ* linezolid, *FICI* fractional inhibitory concentration index.

### Time-kill assays

**Figure 1:**
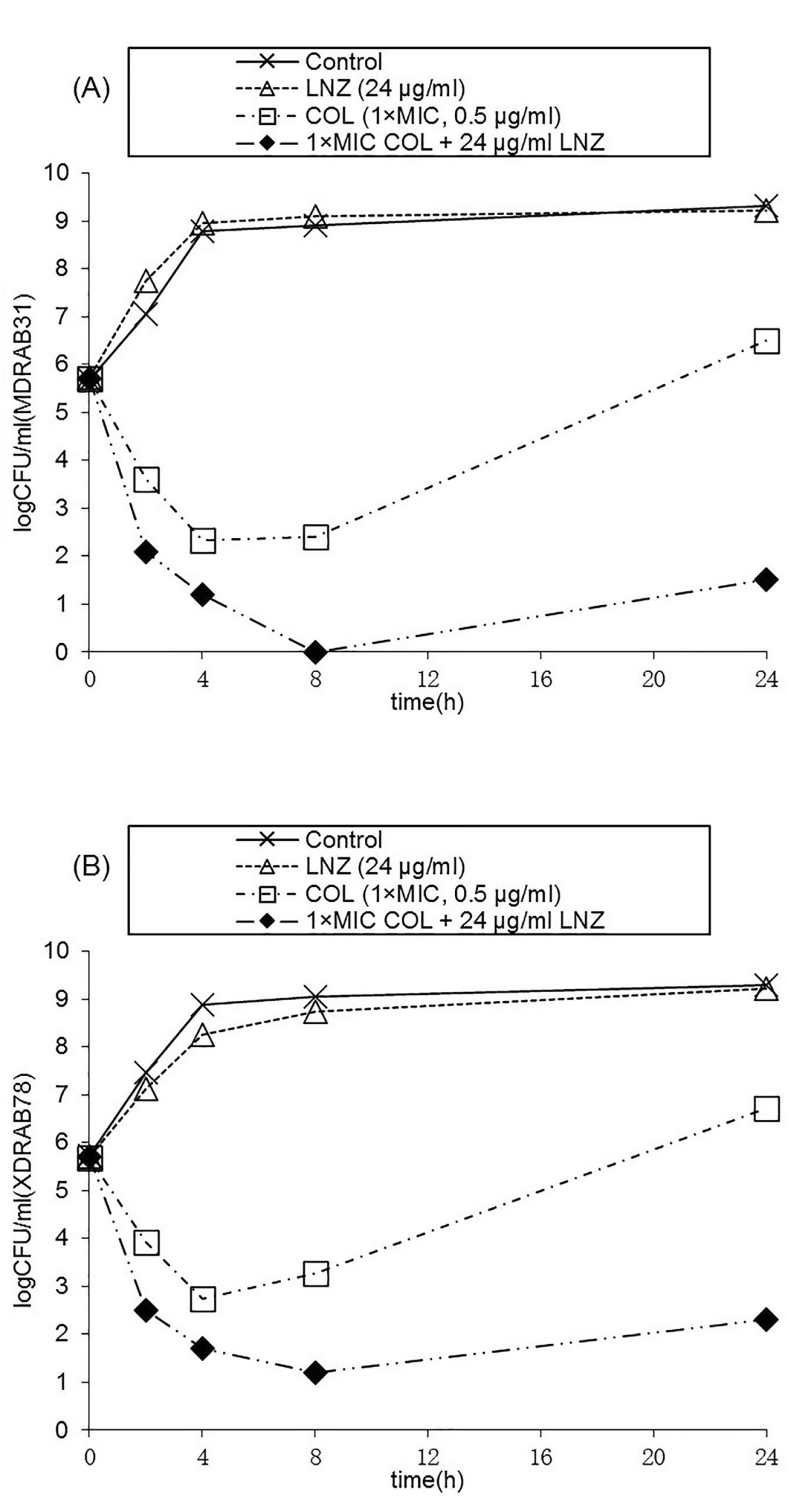
Time-kill curves. Effects of linezolid, colistin and colistin–linezolid combination on the burden for strains MDRAB31 (**A**) and XDRAB78 (**B**). *LNZ* linezolid, *COL* colistin.

Figure 1 shows the time-kill curves of both *A. baumannii* strains. Although bactericidal action against MDRAB31 and XDRAB78 was detected using colistin alone at 0.5 μg/ml (1 × MIC), rapid regrowth was recorded after 8 h. The colistin–linezolid combination showed bactericidal and synergistic effects against MDRAB31 and XDRAB78, with minimal regrowth.

### Survival rates

**Figure 2:**
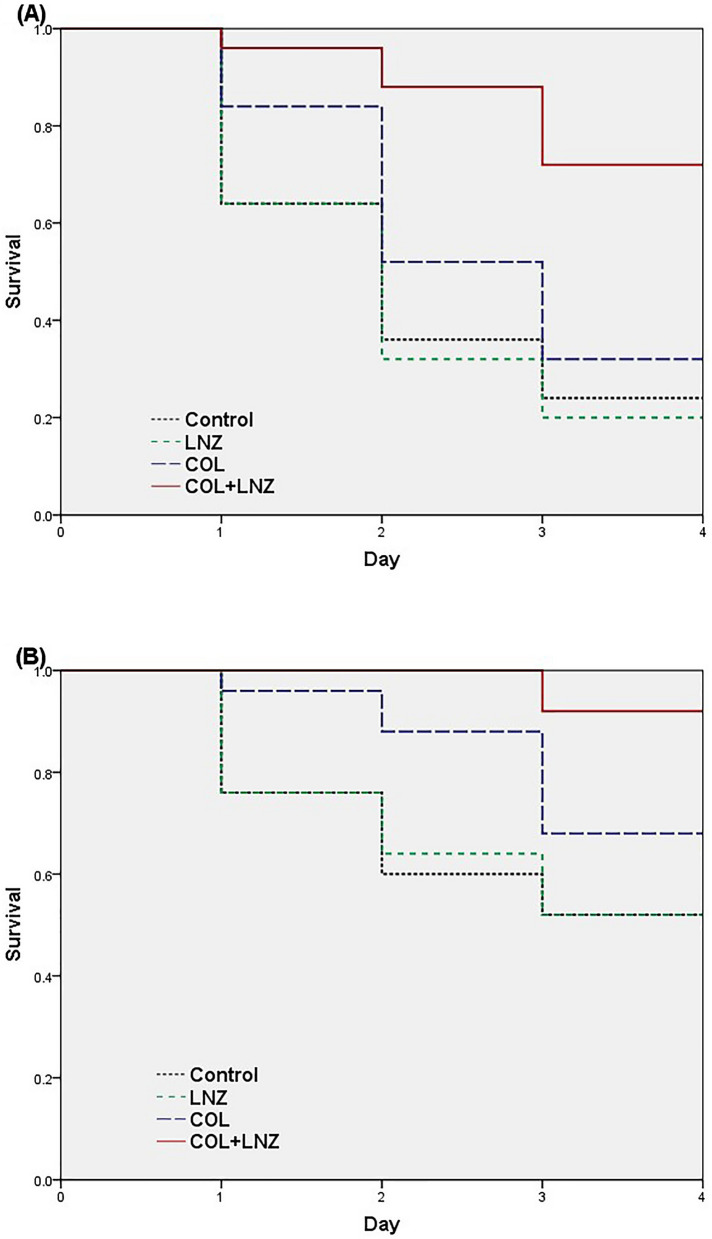
Survival curves. Mice survival with control, linezolid, colistin and colistin–linezolid combination in the murine model of pneumonia for strains MDRAB31 (**A**) and XDRAB78 (**B**). *COL* colistin, *LNZ* linezolid.

The survival curves of both strains are shown in the Fig. 2. For MRDAB31 strain, the survival rates within 4 days were 24% in control group, 20% in linezolid group, 32% in colistin group and 72% in colistin–linezolid combination group. The survival rate of colistin–linezolid combination group significantly increased compared with colistin group (72% versus 32%, *P* = 0.03). Significant differences in survival rates were observed between colistin–linezolid combination group and the other groups (*P* < 0.05).

Comparing with MRDAB31 strain, a lower lethality rate of 48% was observed in control group for XDRAB78 strain. Survival rates of 52%, 52%, 68% and 92% were observed in the control group, linezolid group, colistin group, colistin–linezolid combination group, respectively. There were significant differences in survival rates between colistin–linezolid combination group and colistin group (92% versus 68%, *P* = 0.031), and between the colistin–linezolid combination group and the other two groups (*P* < 0.05).

### Effects on lung bacterial counts

**Figure 3:**
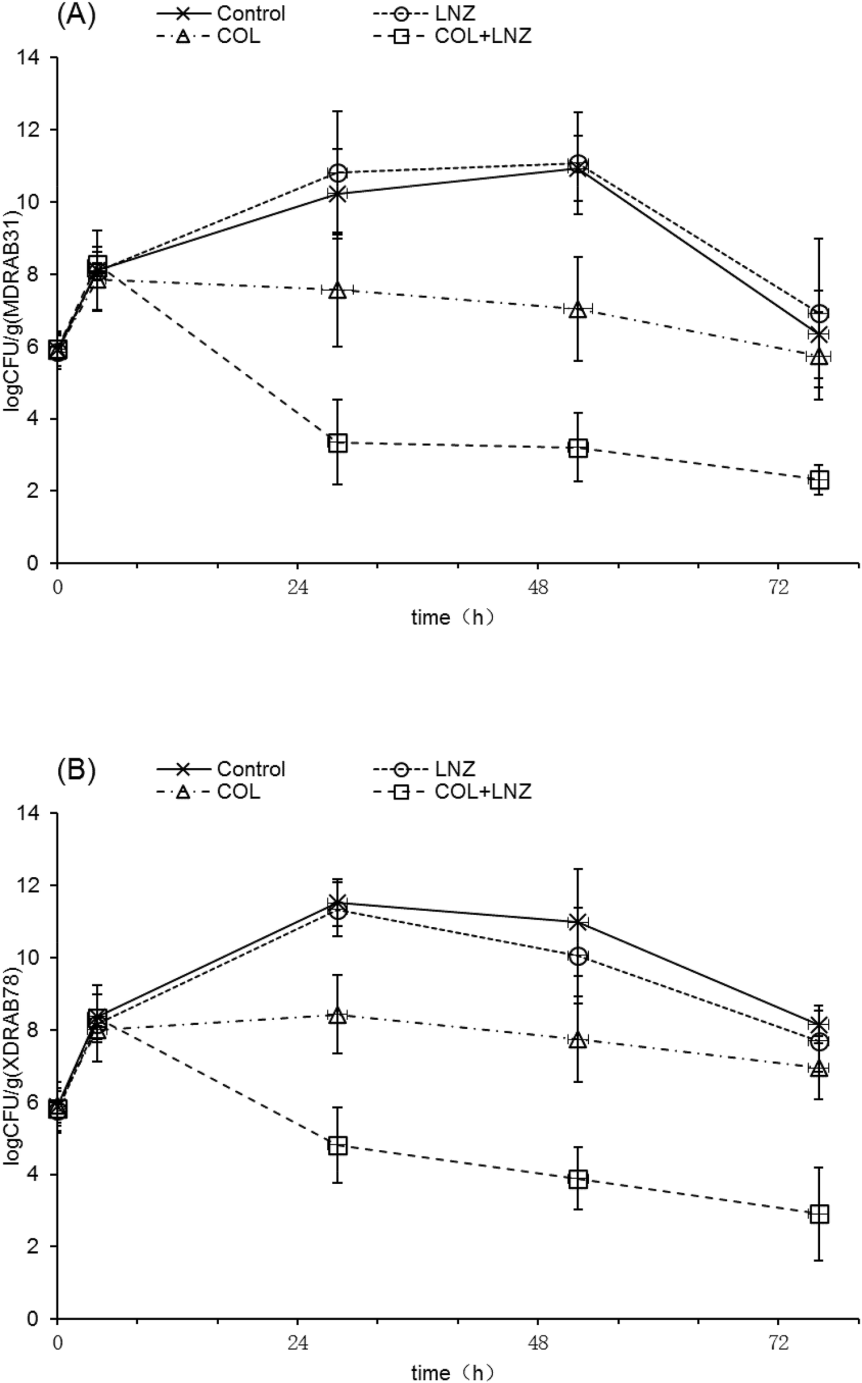
Bacterial counts in lungs (log_10_ CFU/g) with control, linezolid, colistin and colistin–linezolid combination in the murine model of pneumonia for strains MDRAB31 (**A**) and XDRAB78 (**B**). *LNZ* linezolid, *COL* colistin.

Figure 3 shows the evolution of bacterial loads of both strains in each group in lungs after intra-tracheal inoculation. The mean bacterial counts (log_10_ CFU/g of lung) after inoculation 0 h and 4 h were respectively 5.89 ± 0.41 log_10_ CFU/g and 8.08 ± 0.65 log_10_ CFU/g for MDRAB31 strain, 5.84 ± 0.49 log_10_ CFU/g and 8.22 ± 0.57 log_10_ CFU/g for XDRAB78 strain. Colistin monotherapy did not show bacterial effects against both strains. The colistin–linezolid combination groups significantly reduced the bacterial counts in lung tissue compared with colistin groups for MDRAB31 and for XDRAB78 *(P* < 0.05). The colistin–linezolid combination demonstrated bactericidal effects and synergistic effects compared with colistin monotherapy on both strains. The bacterial counts continued to drop, and fell to 2.32 ± 0.41 log_10_ CFU/g for MDRAB31 and 2.91 ± 1.29 log_10_ CFU/g for XDRAB78 at the 76 h.

## Discussion

Our study demonstrated the synergistic activity of colistin–linezolid combination in a murine model of MDR and XDR *A. baumannii* pneumonia for the first time.

Colistin, which was abandoned due to its neurotoxicity and nephrotoxicity, has been reused to fight MDR strains infection^16^. Like in other previous studies^17^, colistin exhibited good activity against *A. baumannii* at first and rapid regrowth was recorded subsequently in time-kill curves. In addition, in Dudhani *et al.* study^18^, similar phenomenon of colistin monotherapy appeared in murine model of *A. baumannii* infection. In 2006, Li *et al.*^19^ defined colistin heteroresistance of *A. baumannii* as the emergence of resistant subpopulations from an otherwise susceptible population. Mutant prevention concentration (MPC) studies and pharmacokinetics (PKs)/pharmacodynamics (PDs) studies revealed that monotherapy of colistin was unable to prevent the development of resistance, and may be substantially caused therapeutic failure^20,21^. Those views may explain the phenomenon of bacterial regrowth in vitro observed here when using colistin-monotherapy.

Linezolid is widely used against most Gram-positive bacteria by binding to rRNA on the 30S and 50S ribosomal subunits to prevent the synthesis of protein^22^. The intrapulmonary concentrations of linezolid in epithelial lining fluid were 64.3 ± 33.1 μg/ml at 4 h and 24.3 ± 13.3 μg/ml at 12 h in healthy volunteers for the recommended dosage regimen (600 mg every 12 h)^23^. And an important part of the application of linezolid was the treatment of pulmonary infection^24^. In this study, the MICs of linezolid in combination were significantly lower than the intrapulmonary concentrations in epithelial lining fluid for the recommended dosage regimen. So it provided the possibility of the linezolid-colistin combination to fight *A. baumannii* pneumonia.

Recently, linezolid was introduced as a colistin-combination option for *A. baumannii* infection basing on the mechanism that colistin exerted a subinhibitory permeabilizing effect allowing the second drug to enter cells^14^. Armengol *et al.*^12^ from biophysical point of view, and Ritcher *et al.*^25^ from physicochemical properties point of view, demonstrated that colistin had a synergistic effect and possibility with linezolid against *A. baumannii* strains. Although all the *A. baumannii* strains were high resistance to linezolid, synergy between linezolid and colistin was observed in checkerboard assay and time-kill assay^15^. In addition, another study in checkerboard assay found when the sub-inhibitory concentrations of colistin were incorporated to colistin–linezolid combination, the MICs of linezolid in combination against all *A. baumannii* strains decreased dramatically, ranging from 4 to 16 μg/ml^13^. And in our time-kill assay and in vivo study, the colistin–linezolid combination also showed bactericidal and synergistic effects. More importantly, there were significant differences in survival rates between colistin–linezolid combination group and colistin group in a murine model of *A. baumannii* pneumonia. Further clinical trials are necessary to confirm this result.

In this study, the result of colistin–linezolid combination for XDRAB78 strain was additive in checkerboard assay, while the result of combination was synergy in time-kill assay, when we used 1 × MIC colistin in combination. Sub-inhibitory concentrations of colistin could increase linezolid uptake in *A. baumannii*, and the accumulation of linezolid was colistin-concentration dependent^12^. Moreover, in the emergence of high concentrations of colistin (7 μg/ml), antimicrobials accumulation was obviously increased in *E. coli*^25^. This may explain above phenomenon and highly lights, even in combination therapies, the importance of optimizing the therapeutic regimen of colistin basing on PK/PD.

Although we did not detect the PKs of the bacterial agents used in this study, antibiotic doses were based on other published studies of same species murine model of pneumonia.

## Conclusion

Colistin–linezolid combination therapy had a bactericidal and synergistic effect in vivo in a murine model of MDR and XDR *A. baumannii* pneumonia.

## Materials and methods

### Strains

Two clinical isolates of *A. baumanni*, which were isolated from two unrelated pulmonary infection patients with bacteremia, were studied. The first (MDRAB31) was tested to be a MDR *A. baumanni* strain (resistant to mezlocillin, piperacillin-tazobactam, cefepime, ceftazidime, ciprofloxacin, levofloxacin) and the second (XDRAB78) was tested to be a XDR *A. baumanni* strain (resistant to mezlocillin, piperacillin-tazobactam, cefepime, ceftazidime, ciprofloxacin, levofloxacin, gentamicin, amikacin, imipenem, meropenem) by the VITEK 2 testing system (bioMérieux, Craponne, France). The quality control stain used as internal standard for each batch of tests was *Pseudomonas aeruginosa* ATCC 27853. All strains were stored separately at − 70 °C in form of powder in airtight vials before being subcultured on containing 5% sheep blood Columbia plates (bioMérieux, Shanghai, China).

XuZhou Central Hospital Ethics Committee approved all experimental protocols, and the methods were carried out in strict accordance with the approved protocols. Informed consent was obtained from all subjects.

### Antibiotic susceptibility tests

The minimum inhibitory concentrations (MICs) of linezolid (Pfizer Inc., NY, USA) and colistin (Sigma-Aldrich, St. Louis, MO, USA) was determined in triplicate using microdilution method, according to the protocol of the Clinical and Laboratory Standards Institute (CLSI)^26^. Because there are no CLSI breakpoint criteria for linezolid against *A. baumannii*, the linezolid breakpoints for *A. baumannii* could not be assessed.

### Checkerboard assays

The synergy testing of linezolid in combination with colistin was assessed by standard checkerboard assay using 96-well microtiter plates^27^. The final concentration range of each antimicrobial varied and based on the MIC of each strain. The concentration of the final bacterial suspension was adjusted to 5 × 10^5^ colony-forming units (CFU) per ml in a 100 ml final volume. The assay was performed in triplicate for each isolate. Interaction between colistin combinations with linezolid was calculated using formula as Odds *et al.* previously described^28^. The interpretation of fractional inhibitory concentration index (FICI) result was as follows: synergy, FICI ≤ 0.5; additivity/indifference, 0.5 < FICI ≤ 4; and antagonism FICI > 4.

### Time-kill assays

Bactericidal activity of both agents and their combinations against each isolate was assessed by standard time-kill assay. The bacteria were diluted to a concentration about 5 × 10^5^ CFU/ml in fresh Ca–Mueller–Hinton broth (MHBCA, Oxoid, Ltd., Hampshire, England). 0.5 μg/ml (1 × MIC) colistin was added and linezolid was added at 24 μg/ml to simulate the lowest intrapulmonary concentrations of linezolid in epithelial lining fluid under standard linezolid dosing regimen (intravenous administration 600 mg/12 h)^23^. Viable bacterial counts were performed at 0 h, 2 h, 4 h, 8 h and 24 h. The definition of bactericidal activity of agents was a ≥ 3 log_10_ reduction in CFU/ml compared with the bacterial concentration of starting inoculum. The definition of synergy was a ≥ 2 log_10_ reduction in CFU/ml for the bacterial concentration of combination compared with the most active agent at 24 h^29^. The experiments were repeated in triplicate on separate days.

### Murine model of *A. baumannii* pneumonia

Healthy female, specific pathogen-free, immunocompetent, 6-week-old female C57BL/6 J mice (Animal Core Facility, Nanjing Medical University, Nanjing, Jiangsu, China) weighing 17–19 g were used for the *A. baumannii* pneumonia model. The mice were housed 5 per cage and had access to chow and drink ad libitum throughout the study. Cyclophosphamide (150 mg/kg of body weight in 0.15 ml) was intraperitoneally injected into the animals to render transiently neutropenic on days 4 and 1 before inoculation. All mice were anesthetized by a mixture of isoflurane and oxygen. Then 50 μl bacterial suspension containing 10^9^ CFU/ml was inoculated by a needle through the nose. Antibiotics were initiated 4 h after inoculation and were administered intraperitoneally.

Experimental Animal Welfare and Ethics Committee of the Nanjing Medical University approved all animal experiments, and the methods were carried out in strict accordance with the approved protocols.

### Study groups

In the first section of the experiment, the mice were randomized into four groups of 25 mice each group for each *A. baumannii* strain. The first group received colistin (125,000 UI/kg, every 6 h; 500,000 UI/kg/day)^30^. The second group received linezolid (50 mg/kg, every 12 h)^31^. The third group received the combination of colistin (125,000 UI/kg, every 6 h) and linezolid (50 mg/kg, every 12 h). The control group received saline (every 12 h). The outcome was observed by survival rate.

In the second section of the experiment, the mice were also randomized into four groups (20 mice in each group): saline, colistin, linezolid, colistin–linezolid combination, for each *A. baumannii* strain. Three mice per group at 0 h, 4 h, 28 h, 52 h, 76 h were euthanized before next dosing and lungs were removed for quantitative bacteriological studies. Lungs were weighted and then homogenized in 1 ml of saline. 0.01 ml serial tenfold dilutions of homogenates were plated on containing 5% sheep blood Columbia plates at 37 °C for 24 h. The definition of bactericidal activity of agents was a ≥ 3 log_10_ reduction compared with the bacterial concentration before first dosing. The definition of synergy was a ≥ 2 log_10_ reduction in CFU/g for the combination compared with the most active single agent^32^.

### Statistical analysis

The data were presented as the means ± SD (standard deviations). Log-rank test and Kruskal–Wallis test were used to compare survival and bacterial counts in lung tissue respectively between groups of each strain. In all experiments, statistical significance was accepted when the *P* value was < 0.05.
